# Post-breeding movements of Ancient Murrelet *Synthliboramphus antiquus* family groups, subsequent migration of adults and implications for management

**DOI:** 10.1371/journal.pone.0171726

**Published:** 2017-02-24

**Authors:** Anthony J. Gaston, Yuriko Hashimoto, Laurie Wilson

**Affiliations:** 1 National Wildlife Research Centre, Science and Technology Branch, Environment Canada, Ottawa, Canada; 2 Canadian Wildlife Service, Environment Canada, Pacific Wildlife Research Centre, 5421 Robertson Rd, RR#1 Delta, British Columbia, Canada; University of South Carolina, UNITED STATES

## Abstract

Increased shipping in British Columbia (BC) waters poses risks for marine birds from marine oil spills. Ancient Murrelets (*Synthliboramphus antiquus*), small marine diving birds of which half of the world’s population breeds in BC, are especially susceptible to oiling immediately after departing from their breeding colonies, as their offspring are flightless, constraining their parents to remain with them. In 2014 we deployed geolocator loggers on Ancient Murrelets at four breeding colonies, two on the east and two on the west coast of Haida Gwaii to investigate patterns of post-breeding dispersal and subsequent migratory movements. Birds from east coast colonies moved south and east after leaving their colonies, remaining in Queen Charlotte Sound and adjacent waters for 4–6 weeks, whereas those from west coast colonies moved steadily north and west, so that they left BC waters earlier than those from east coast colonies. These movements were consistent with being driven by surface currents. In June, all birds moved rapidly to the eastern Aleutians, SE Bering Sea, and waters off Kamchatka, where they probably moulted. In August, most moved north, some passing through Bering Straits into the Chukchi Sea. In October-November some birds returned to waters off western N America (33%) and the remainder carried on westwards to waters off Japan, Korea and NE China. For the former group the movement to the Bering Sea in June constituted a moult migration and, as such, is the first described for an auk. Those birds wintering in Asia began moving east in February and arrived off BC in March, when observations at colonies show that burrow visits begin in Haida Gwaii. Our data suggest that, immediately after colony departure, birds from the east coast colonies (about half the population of Haida Gwaii) are at higher risk from potential oil spills in northern British Columbia waters than those breeding on the west coast.

## Introduction

“*The Ancient Murrelet cannot be described as a migrant*, *in the sense of having clearly defined wintering and summering areas*.” [1]

The transport of hydrocarbons through the marine waters off northern British Columbia has been the subject of public debate for many years [2,3]. Given the nature of marine transport, there is an inevitable possibility of oil spills consequent on tanker accidents (catastrophic spills), or deriving from routine operations (chronic spills) [3]. To evaluate the potential consequences of any such spills, we need to know the vulnerability of different marine organisms, especially those where a large proportion of the population is concentrated within the potential spill area. Vulnerability depends on where a species spends time, on how much time is spent in each area, and on the behaviour of the organism, which determines the likelihood that it will contact the oil [4].

The Ancient Murrelet *Synthliboramphus antiquus* is a small diving bird of the Auk family (Alcidae). It is unusual in having nestlings which leave the breeding burrow within 72 h of hatching and go to sea, being accompanied and fed by both parents for several weeks after departing from land [1,5]. During this period, the chicks are incapable of flight, so the family party is necessarily confined to swimming. Sightings of chicks at sea after colony departure generally involve family parties including two adults [5,6,7], so it is assumed that both parents accompany the chicks for a period of several weeks. There is no evidence that the adults molt during this period [8].

The majority of Ancient Murrelets breeding in North America south of Alaska, amounting to about half of the world population, nest in the Haida Gwaii archipelago, British Columbia [9]. The Ancient Murrelet is listed as a “Species of Special Concern” by the Committee on the Status of Endangered Wildlife in Canada, mainly because of reductions in population consequent on the depredations of rats *Rattus* spp. and raccoons *Procyon lotor* introduced to their nesting islands in Haida Gwaii in the 20^th^ century [10,11]. The species is observed in winter at sea in British Columbia waters, as well as waters south to California. However, recent information from geolocator tracks of four birds breeding in Haida Gwaii showed that all four moved west to the Bering Sea after the completion of breeding and at least three of them continued westwards to winter off China, Korea and Japan [12]. All had left Canadian waters by the end of July and observations at sea suggest that the species is almost entirely absent from BC waters during August and September, re-appearing in October [9].

Movements of family parties in the first few days after departure from land were described for birds from one Haida Gwaii colony [13]. There have been scattered sightings of Ancient Murrelet family groups during the post-departure period throughout Hecate Strait, Queen Charlotte Sound and waters off the west coast of Haida Gwaii [6,7,14], but little is known about the post breeding dispersal of family parties, or exactly when the adults begin to shift to Alaskan waters and when they return. At-sea observations to date provide only imprecise information on the presence and distribution of Ancient Murrelets in BC waters because of limited coverage and replication, although most observations have been made either on the continental shelf or close to the shelf break [15,16].

In the event of an oil spill, Ancient Murrelet family parties which had just departed from their colonies, being confined to moving on the surface, would be highly vulnerable to contamination. In order to assess the degree of risk and the colonies most likely to be affected, the Canadian Wildlife Service of Environment and Climate Change Canada deployed a number of geolocator devices on breeding Ancient Murrelets at four breeding colonies in Haida Gwaii during the 2014 breeding season. This paper analyses the movement of birds during the immediate post-departure period and assesses the extent to which they may intersect with proposed oil tanker routes within the Canadian Exclusive Economic Zone (within 200 nm of the coast). Subsequent movements (after passing westward of 140 W) which are not relevant to an assessment of potential oil spill effects in BC waters, are also described in outline. However, although most birds wintered off Asia, as described by Gaston et al. [12], a minority (33%) returned to the west coast of Canada and the United States in October and wintered there. We also consider here the distribution of those birds in winter in relation to proposed tanker routes in BC waters. We do not deal with the distribution of Ancient Murrelets during the breeding season (mid-March to mid-May [1]), as during that period they have to return to their breeding sites every few days and colony locations are well known [1,17].

## Methods

### Permits

Our project was approved, in 2013 & 2014 by the Simon Fraser University Animal Care Committee (protocol #974B-94), and in 2015 by the Environment and Climate Change Canada Western & Northern Region Animal Care Committee (project #15LW01).

The following permits were obtained:

BC Ministry of Environment: Ecological Reserve Permit #107132 (for Hippa); Park Use Permit Research Permit #106604 (for Frederick & Reef islands)

Parks Canada: Parks Canada Research & Collection Permit #GWA-2013-13715 (for George)

Environment and Climate Change Canada: Scientific Permits: 2013: #BC-13-0018, 2014: #BC14-0026, 2015: #BC-15-0008); Banding Permits (Canadian Wildlife Service): Laurie Wilson 10667J, Dan Shervill 10667K, Glen Keddie 10067L, Jake Pattison 10425AB".

### Field work

**Fig 1:**
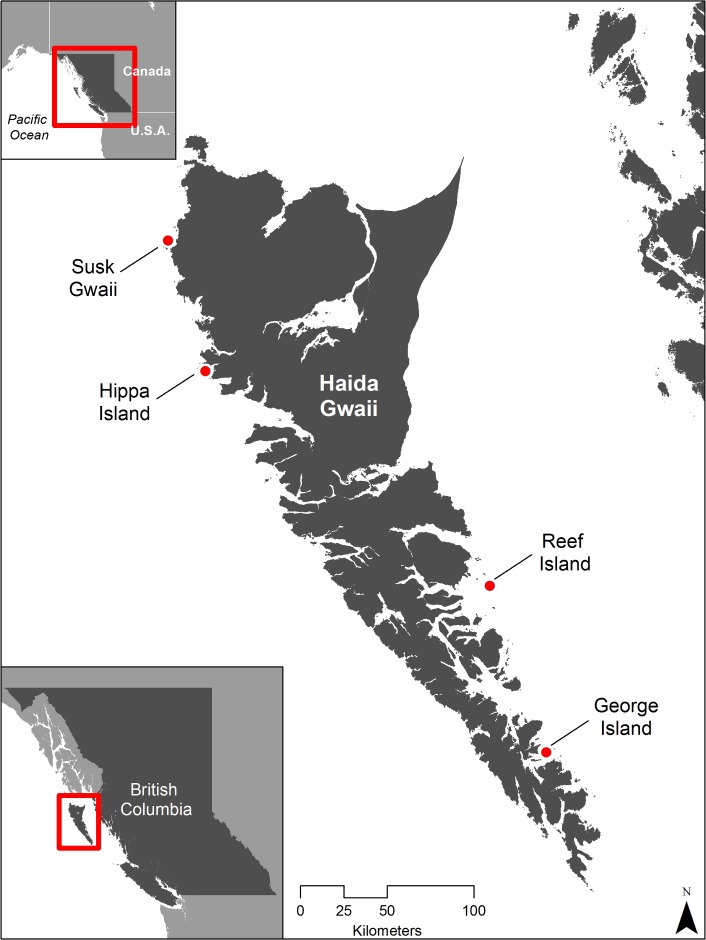
Map of colony locations.

Geolocators were deployed on breeding Ancient Murrelets at four islands in the Haida Gwaii archipelago: Hippa and Susk Gwaii (west coast) and George and Reef (east coast)(Fig 1). Deployments were timed to take place in the second half of incubation, at which point breeders were considered less liable to desert their eggs than earlier in incubation (Table 1). A geolocator (Intigeo-C65, Migrate Technology, Cambridge, UK) was secured to a metal band using a small black tie or 30-lb test spiderwire and mounted on the right leg. The total weight of the geolocator and band was 1.7 g, <1% of adult body mass (220 g, [1]) and device dimensions were 14 x 8 x 6 mm. Burrows at which the geolocators were deployed in 2014 were revisited in 2015. If the geolocator-tagged adult was present, the tag was removed. Where a non-tagged adult was present, a few twigs were set up to lightly block the burrow entrance. Burrow entrances were monitored daily and, when twigs were disturbed, indicating a possible change-over, the burrow was checked for a new adult.

**Table 1:** Dates of deployment of geolocators on Ancient Murrelets in 2014, and numbers deployed and recovered with usable information, by nesting island (excludes 2 birds for which the geolocators yielded no data).

Coast of Haida Gwaii	Breeding colony	Geolocators deployed in 2014	Median deployment date in 2014 (range)	Geolocators recovered in 2015	Median date of recovery in 2015 (range)
West	Susk Gwaii (Frederick I.)	50	13 May (12–14 May)	20	14 May (13–23 May)
	Hippa I.	25	9 May (9–10 May)	4	14 May (12–15 May)
East	George I.	50	1 May (30 Apr- 2 May)	18	5 May (1–8 May)
	Reef I.	25	1 May (1–2 May)	7	2 May (2–7 May)
	Combined	150	30 Apr—14 May	49	1–23 May

When the geolocator was removed, the adult was also weighed, six feathers were hand plucked from the breast, and a small blood sample (1 ml) collected from the brachial vein using a 27-1/2” gauge needle and 3 ml syringe, and stored in a vacutainer with sodium heparin. Two small circles of whole blood were dotted on filter paper (~ 10 ul blood/circle) (Protein Saver Snap Apart, Whatcom 903, GE Healthcare), air dried and stored at room temperature. A small amount of formalin (ratio 1:20, formalin:blood) was added to the remaining blood to preserve the sample. Filter paper samples were used to determine sex from DNA using PCR via test method MET-DNA-SEX-01C, at the National Wildlife Research Centre, Environment and Climate Change Canada, Ottawa

### Data processing

Geolocator data were downloaded and processed with IntiProc v1.03 [18]. Sun elevation angles used to estimate sunrise and sunset were calibrated based on light levels recorded when birds were at-sea in the post-deployment and pre-recovery incubation periods during which birds were assumed to be near the known deployment sites. Estimated locations during incubation periods were an average geodesic distance of 177 ± 97 km (about 2 degrees) from the deployment/recovery sites. Due to unknown shading conditions affected by bird behavior and anatomy, changes to light sensor housing and environmental factors [19] the elevation angles used are a best approximation. During spring and fall equinoxes day length is similar throughout the globe, producing uncertain latitudinal estimates. Latitude estimates between 22 September—27 October 2014 and 23 February—30 March 2015, when latitudinal variance was high, were excluded from most analyses. Points east of 115° W and west of 120° E, or with latitudinal values south of 20°N were also excluded, as being outside the known range of the species, but were included in figures for March and October, because their exclusion would have resulted in very few points for those months and longitude estimates are unaffected by the equinox effect [20]. Points were mapped and analysed in ArcGIS Desktop 10.1 [21].

### Data selection and analysis

Ancient Murrelets do not remain in their breeding burrows during the day until they begin incubation. Thereafter, the members of breeding pairs alternate incubation shifts, which generally last 2–5 days [9]. Consequently, incubation behaviour can be identified from geolocator data based on the first date when no light was recorded (start of first incubation shift for that individual) and the last date when no light was recorded (following day is first day post-departure). One bird departed the nesting island on 2 May (first day after deployment), earlier than any previously recorded departures among thousands of departures monitored in previous years [1]. Three other birds departed the day after the logger was deployed. It seems likely that these four birds deserted their clutches and that subsequent movements were not representative of a family party, so we have omitted them from analyses of movements during the period immediately after colony departure. The response variable date of burrow departure, date of start of incubation and date of beginning westwards movement (last date when the individual was east of 140° W) were analysed in relation to sex, breeding island, breeding coast (E or W coast) and wintering zone (Asia, N America, based on their position on 1 January: E or W of the international date line) using one-way ANOVA and Duncan’s Multiple Range test for post-hoc differences. Dates of the start of incubation in 2015 were compared with dates of departure in 2014, breeding coast and wintering zone using GLM.

Mean values are given ± SD. All variables were tested for normality using Lilliefor’s test and non-parametric probability values were used where data were not normally distributed. For non-parametric comparisons of means we used Mann-Whitney U tests and to estimate 2 x 2 probabilities we used the Fisher exact probability test (2-tailed). All statistics were calculated using Statistica 7.0 [22].

## Results

### Pre-fledging period

After excluding the four birds which apparently deserted immediately after logger deployment, the date of mean colony departure was 25 May ± 8 days (N = 45), within the range of median values reported over five years at Reef Island [1], with departures being significantly earlier at Reef Island than at Susk Gwaii (Table 2).

**Table 2:** Mean dates of burrow departure in 2014, of last day to the east of longitude 140 W, of first day to W of longitude 160 W, mean duration of time from burrow departure to the last day E of 140 W and mean date of the start of incubation in 2015.

Breeding island	Mean date of departure from burrow, 2014 (n)	Mean last date E of 140°W	Time from departure to last day E of 140°W (days)	Mean last date in 2015 W of 140°W (range)	Mean date of beginning incubation, 2015 (n)
Susk Gwaii	30 May* (18)	25 June	33	20 March (9–31 Mar)	4 May* (18)
Hippa	25 May (4)	6 July	49	22 March (15 Mar– 1 Apr)	1 May (4)
George	21 May (16)	24 June	36	20 March (10–31 Mar)	24 April (17)
Reef	20 May* (7)	27 June	41	19 March (15–27 Mar)	17 April* (7)
Combined	25 May (45)	25 June	37	20 March (9 March– 1 April)	28 April (46)

*colonies differing at P<0.05 in dates of burrow departure in 2014 (col 2) and dates of starting incubation in 2015 (col 6), based on one-way ANOVAs and Duncan Multiple Range Tests

**Fig 2:**
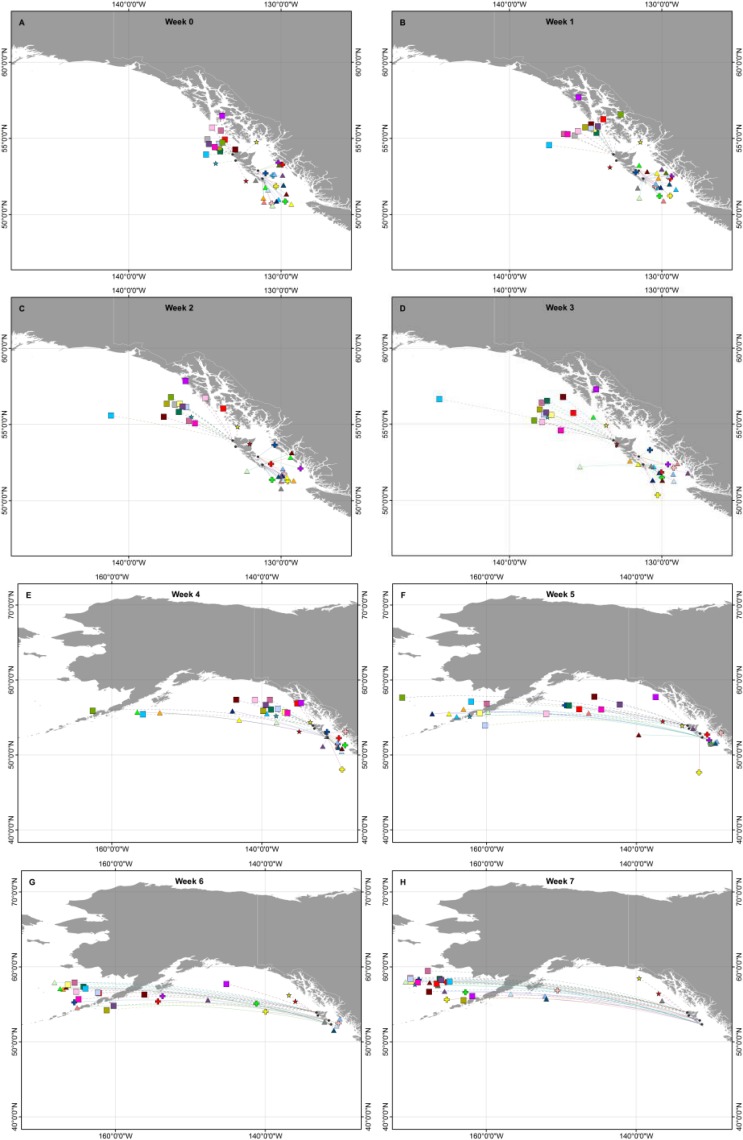
Weekly mean positions of individuals tracked during the period from 0–7 weeks after colony departure. Symbols: squares, Susk Gwaii; stars, Hippa Island; crosses, Reef Island; triangles, George Island.

Movements of birds from east coast colonies during the first week after departure were necessarily eastwards into Hecate Strait. The majority subsequently moved south into Queen Charlotte Sound, a few apparently hugging the mainland coast, while others shifted westwards to waters off northern Vancouver Island (Fig 2A–2H). Birds from the west coast colonies, by contrast, moved mainly north and west after departure from the colony with a few probably entering waters within the SE Alaskan archipelago. Birds from east and west coast colonies were completely segregated until four weeks after colony departure, except for those birds passing westwards across 140°W.

**Fig 3:**
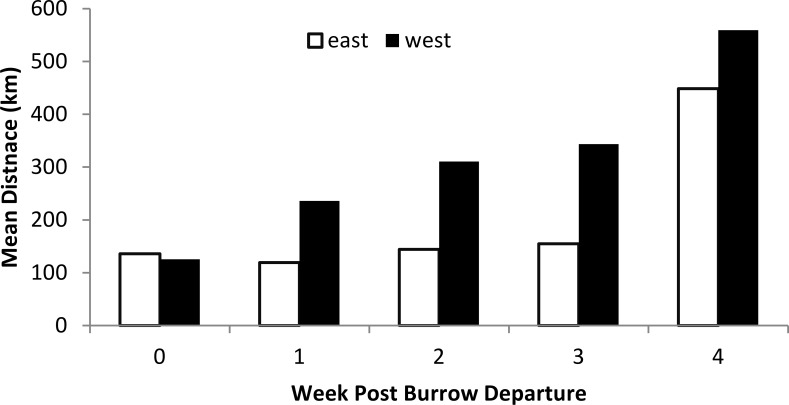
Mean geodesic distance from the colony during the first five weeks after colony departure for colonies on east and west coasts of Haida Gwaii.

The rate of dispersal away from the colony differed between east and west coasts, with birds from the west coast moving steadily further from the colony during the first four weeks after departure, while those from the east coast travelled a similar distance during the first week but then remained at more or less the same distance from the colony for the next three weeks (Fig 3).

### Initial migration

**Fig 4:**
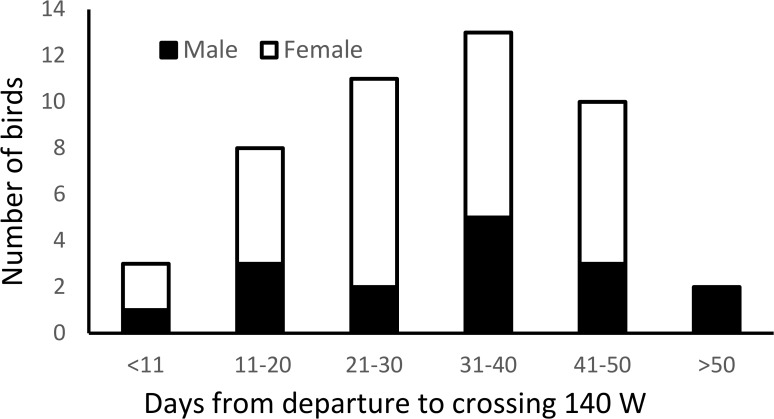
Time from burrow departure to the last day east of longitude 140° W.

**Fig 5:**
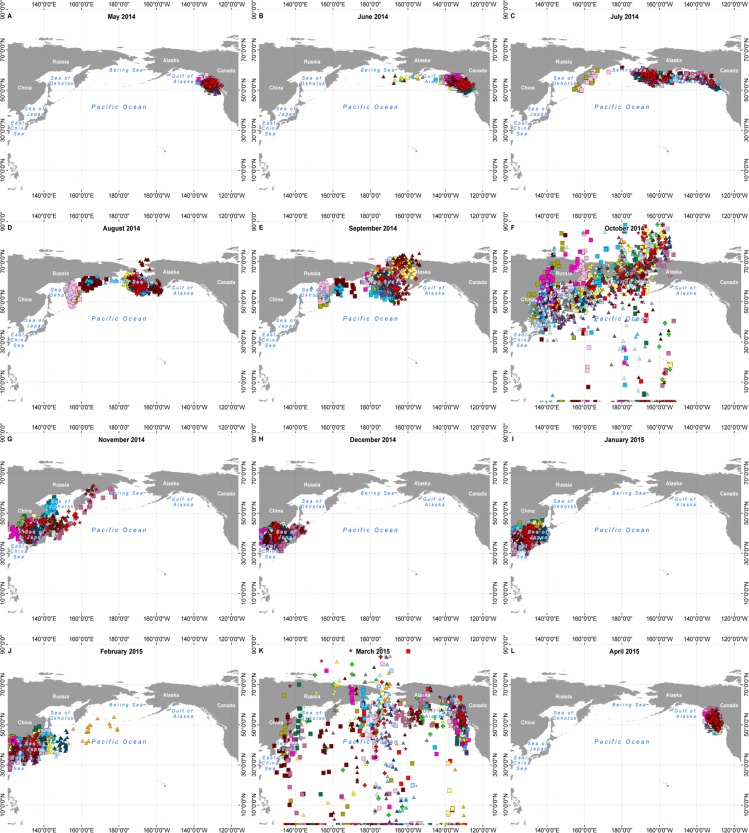
Daily positions of individual Ancient Murrelets wintering off Asia (n = 24), by month, May 2014 –April 2015. Only individuals thought to have departed successfully with their chicks and for which the initiation of rapid westwards movement took place at least 21 days after burrow departure are included, as all these birds should have successfully reared chicks to independence (see Discussion). Colour and symbol codes apply to the same individuals as Fig 2. All maps were filtered according to the criteria given in the Methods, except for October (f) and March (k) where the great spread of latitude would have meant very small samples after filtering. These maps are included to illustrate the spread of longitude in those months. An explanation for the large latitude spread during the months of October and March is given under Methods/Data processing: see also [20].

**Fig 6:**
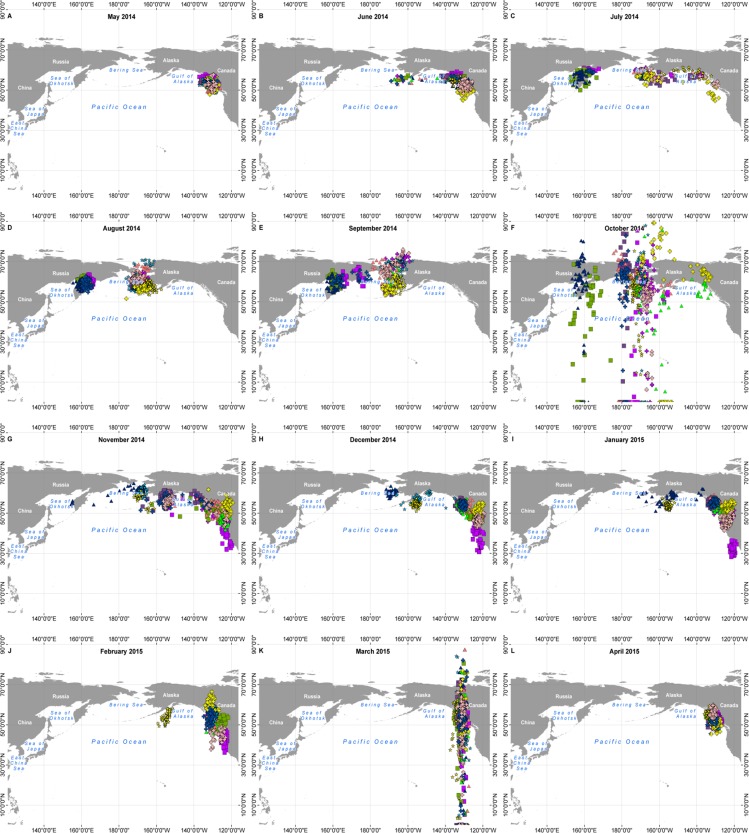
Daily positions of individual Ancient Murrelets wintering off North America (n = 11), by month, May 2014 –April 2015. Colour and symbol codes apply to the same individuals as Fig 2; data selected and filtered as for Fig 5.

Rapid westward movements began 9–65 days after burrow departure (mean 32 ± 13 days, Fig 4) and did not differ among islands (ANOVA, F_3,42_ = 2.06, P > 0.1) or between coasts (t_44_ = 1.00, P > 0.1) or sexes (t_44_ = 0.50, P > 0.1). Ten birds (6 from west coast colonies, 4 from east coast colonies) spent less than 21 days after burrow departure before moving rapidly westwards. The duration of time between burrow departure and crossing longitude 140° W was negatively correlated with date of burrow departure: birds leaving the colony later spent less time before starting to move westwards (Pearson r_44_ = 0.55, P < 0.01). Movements after crossing 140°W were rapid, reaching 160°W a mean of 7.0 ± 4.5 days later (1300 km at 54° N), with all but six birds (all from west coast colonies) taking less than 10 days (Table 2). All birds were in waters to the west of 150° W (central Gulf of Alaska) by mid-July and remained to the west of that longitude throughout August and September (Figs 5 and 6).

In July and August, two concentrations of birds were clearly discernable (Figs 5 and 6), the larger one clustered around the Alaskan Peninsula, eastern Aleutian Islands and eastern Bering Sea (eastern cluster, 40 birds); the other situated farther west, around the coast of Kamchatka and at the northern end of the Sea of Okhotsk and Shelikov Gulf (western cluster, 9 birds). In the latter half of August and more obviously in September, most birds in the eastern cluster moved into the northern Bering Sea and at least ten birds seem to have moved through Bering Strait (presumably) to the Chukchi Sea and adjacent parts of the Arctic Ocean.

### Migrations post-moult

In October and more commonly in November, some birds returned to the west coast of North America between SE Alaska and California, while the rest continued westward to Asian waters (Fig 4). All those in the western cluster in August continued towards the southwest to winter off Japan and Korea except for one which returned eastwards in October and early November and which, by the end of December, was off southern California. Of the eastern cluster, 28 continued to Asian waters, while 12 returned to the west coast of North America (east vs west cluster, Fisher exact test P = 0.66). Among those which passed through the Bering Strait, 3 returned to North America, while 7 spent the winter in Asia. In November (Fig 6G), a cluster of five birds aggregated in the western Gulf of Alaska for a period before continuing to British Columbia and southwards. By December (Fig 6H) only three birds remained in Alaskan waters.

### Wintering

By January, wintering areas were well-defined and widely separated, with 33 birds in Asian waters on 1 January and 16 in waters off North America, of which two were in Alaskan waters and the rest in waters from British Columbia south to California (Fig 6I). Birds wintering on the Asian side of the Pacific did so mainly in the Sea of Japan and as far west as the Yellow Sea (Fig 5I). One bird returned from Asian waters in midwinter, passing eastbound across 140° W on 25 January 2015. The remainder of birds wintering off Asia started their eastward migration in February and early March and the average date of passing eastbound across 140° W was 21 March for both males and females (range 7 March– 3 April 2015). There was no difference among colony locations in the proportion of birds going to Asia, forming part of the east or west clusters in August, or moving north into the Chukchi Sea (Table 3, all Fisher exact test P > 0.1). Nor was there any difference in the date of moving westward across longitude 140° W between birds which continued to Asia and those which came back to winter off N America (t_45_ = 1.51, P = 0.14).

**Table 3:** Numbers of birds from east and west coasts of Haida Gwaii in either eastern or western moulting clusters (August positions), numbers visiting the Chukchi Sea, north of Bering Strait (September) and wintering in Asia or North America (January).

Colony region	Moulting Cluster		Visiting Chukchi Sea		Wintering	
	Western	Eastern	Yes	No	Asia	N America
East coast	3	22	6	19	18	7
West coast	6	18	4	20	15	9
Fisher exact P	0.29		0.72		0.55	

Birds which wintered in North American waters mostly left the Bering Sea and Gulf of Alaska in October and November, but a few remained through December and one lingered until February (Fig 6J). By December, most birds off North America were centred between 45–55°N, from northern Oregon to northern British Columbia, but two were as far south as Central California (35°N). They remained in the same region until February. In March, although latitude was uncertain because of the equinox effect, their longitudes showed them probably in the region of Haida Gwaii (Fig 6K).

### Initiation of incubation

**Fig 7:**
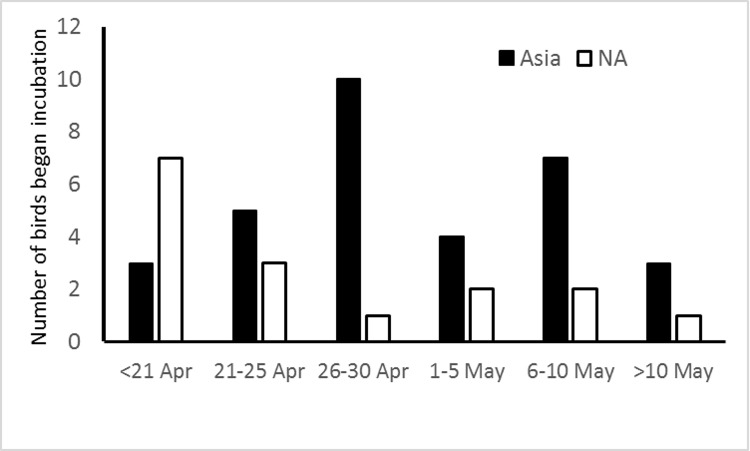
Distribution of dates of initiation of incubation by geolocator-equipped Ancient Murrelets in 2015 comparing wintering areas: waters off Asia (Asia) and North America (NA).

All birds were within 500 km of their banding colonies in April (Figs 5L and 6L). The mean date at which incubation began in 2015 was 28 April ± 9 days (N = 46), with similar variation among colonies to that seen in 2014 dates of colony departure (Table 2). Birds which wintered in Asian waters began incubation, on average, significantly later than birds wintering off North America (Mann-Whitney U = 130.5, 2-tailed exact P = 0.015; Fig 7), although the spread was similar. When breeding coast and wintering area were both entered as independent variables in an ANOVA for date of start of incubation only wintering area was found to have a significant effect on date of starting incubation (F_1,44_ = 5.30, P = 0.03).

## Discussion

Timing of burrow departure by the tracked birds was similar to reports from other years [1]. Laying and hatching tend to be later on the west coast of Haida Gwaii than on the Hecate Strait side [23] and this was reflected in our samples. The similarities suggest that most birds equipped with loggers departed normally along with their chicks.

We do not know whether chicks accompany their parents on the westward migration to the Bering Sea in June and July, but in any case, they would need to be able to fly in order to do so. Judging from the speed of feather development in other auks (shortest nestling period in the fastest growing species, Least Auklet *Aethia pusilla* and Little Auk *Alle alle*, 23 days [24]), chicks probably would not be able to fly until at least three weeks after leaving the colony. Hence, the eleven birds which began rapid westward movement less than three weeks after departing from their breeding colony had either deserted their eggs, or their chicks had died. Some losses during the immediate post-departure period seem likely, especially if weather is persistently bad. The breeding pairs cannot delay their departure from the burrow by more than 1–2 days without endangering the chicks, which are not fed at that period [4].

Southward movement of birds from the east coast colonies into Queen Charlotte Sound has been inferred previously by Sealy et al. [7] and is supported by surveys carried out by Raincoast Foundation in 2008, when densities of family groups were high in Queen Charlotte Sound [25]. Conversely, the northwesterly movement of birds from west coast colonies was not previously known.

Given the lack of evidence for Ancient Murrelets moulting in British Columbia waters [8] it seems unlikely that moult would begin until after the westward migration in June. This must occur after chicks are flying, so the fact that 74% of birds crossed 140 W within 40 days of leaving the colony suggests a fledging period of <6 weeks. This is consistent with the appearance of independent young in early July [8] and suggests a post-departure fledging period similar to that of young murres (*Uria* spp.)[22]. Birds collected off Vancouver Island in October were in freshly moulted plumage [9] and observations of birds off St Lawrence Island and on shipboard surveys of the northern Bering and Chukchi seas in August and September suggested that most were in basic plumage at that season (R. Day, A. Gall, J. Plissner and G. van Vliet personal communication). Out of sixteen adult Ancient Murrelets for which plumage was noted on shipboard surveys in the Bering Sea north of 63° N in late August and September 2013, all were in basic plumage (K. Kuletz, E. Labunski unpubl.). This evidence suggests that pre-basic moult mostly takes place during July-August, in which case, for birds originating from Haida Gwaii, it almost certainly occurs in the cluster areas: southeast Bering Sea and off Kamchatka. For the birds returning to winter off North America the westward movement to the Bering sea is clearly a moult migration [26] and constitutes the first such dedicated moult migration described for any auk.

**Fig 8:**
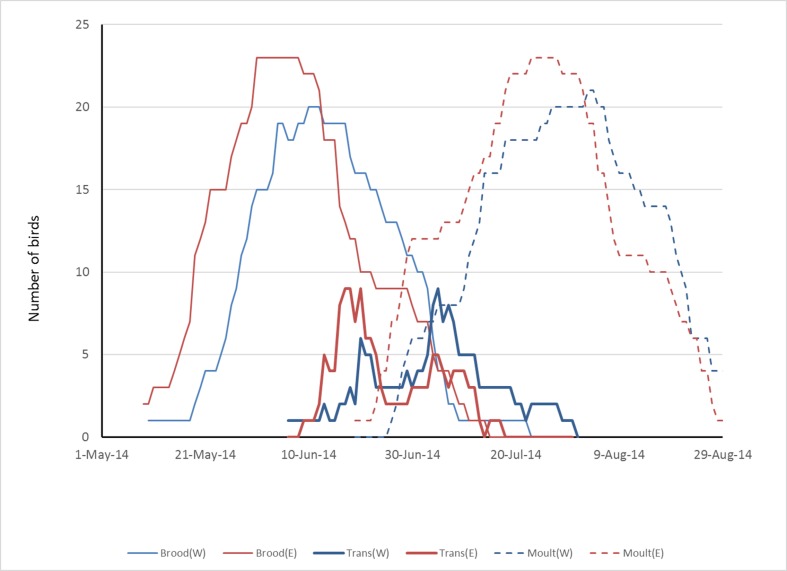
Projected numbers of logger-equipped Haida Gwaii Ancient Murrelets from west (W) and east (E) coast colonies assumed to be engaged in brood-rearing (brood), rapid westward movement (transit) and moult during May-September 2015.

The timing and duration of moult is not well known in auks, but is believed to be about 6 weeks for a complete primary moult in most species [24]. If moult begins soon after birds arrive in the southeast Bering Sea and off Kamchatka, then a six-week moult would fit into the period prior to northward movement in Bering Sea. Assuming that moult begins five days after crossing 160 W (arriving on the moulting grounds) and takes 40 days to complete then the peak of moult for the Haida Gwaii population would have fallen between approximately 15 July and 20 August in 2014 (Fig 8): a good fit with the presence of birds in basic plumage in northern Bering Sea in September.

Segregation of birds from east and west coast colonies was most marked during the immediate post departure period, with most of those from east coast colonies spending the first 4–5 weeks after departure in Hecate Strait and Queen Charlotte Sound, whereas birds from west coast colonies spent that period principally offshore north and west of Haida Gwaii. Currents in Hecate Strait and Queen Charlotte Sound in May are generally tidal without a strong unidirectional component, although there is a weak northward drift [27]. This would tend to maintain flightless family parties within Hecate Strait and Queen Charlotte Sound, accounting for the fact that they do not appear to move away from their colonies after the first week (Fig 3). Conversely, family parties moving westwards from the west coast colonies would encounter the relatively constant flowing Alaskan Current [28] which would carry them steadily north and westwards: exactly the predominant direction observed during the first 2–3 weeks after departure from the colony. Consequently, the apparent divergence in behaviour between east and west coast colonies during the immediate post-departure period may be a passive response to local geography and oceanographic conditions, rather than a deliberate difference in strategy.

Because of the error inherent in the light geolocator loggers [29] we cannot be sure whether birds from the east coast colonies passed through Dixon Entrance, or to the west of Haida Gwaii during their Alaska-bound migration, but the paucity of locations in Dixon Entrance suggests that any which used that route passed through quickly. Once west of 140° W there was broad overlap of birds from different colonies in July and August, and this continued through the winter, with similar proportions from both coasts of Haida Gwaii moving to Asia or returning to winter off North America. The behaviour of birds subsequent to their westward migration in June shows no variation among colony locations or sexes, showing that the different migration strategies, especially the divide between Asian and North American wintering areas, are not colony-specific.

Multiple discontinuous wintering areas are known for many species of birds and are particularly characteristic of those which have colonised their breeding grounds relatively recently, such as King Eider *Somateria spectabilis* and Sabines’s Gull *Xema sabini* [30, 31]. However, in most cases, individuals adopting different wintering areas derive from different parts of the breeding range [32]. In the case of Ancient Murrelets breeding in Haida Gwaii, we found no separation among breeding colonies of birds wintering off Asia or off North America. The only comparable cases hitherto documented for seabirds appear to be those of Sooty Shearwaters *Puffinus griseus* breeding at two sites in New Zealand which use one of three discrete wintering areas in the North Pacific [33] and Cory’s Shearwater *Calonectris diomedea* breeding on the Azores, the Canary Islands and in the Mediterranean, which intermingle on four discrete wintering areas in the Atlantic [34]. However, no common moulting area is known in either case.

Movements once the adult birds were independent of their offspring, which began for all birds within about eight weeks of colony departure, consisted of periods of rapid travel interspersed with relatively sedentary periods (“oriented chain migration” as per Fort et al. [35], also “stepping stone migration” [36]). In June, passage across the Gulf of Alaska was rapid, but was followed by 2–3 months either in the eastern Aleutians and eastern Bering Sea or off the coast of Kamchatka. In October and November, the migratory divide appeared: the majority moved west to the Sea of Okhotsk and waters around Japan while about one third moved back to the western Gulf of Alaska and coastal waters from British Columbia to California. Distributions were relatively static in December and January, followed by a very rapid eastward return from mid-February to mid-March, as identified by Gaston et al. [12]. The migration pattern of the Ancient Murrelet can therefore be regarded as consisting of a local post-breeding dispersal followed by a movement to the presumed moulting areas in the eastern and western Bering Sea, a shift to wintering areas either off the coast of North America or Japan and Korea and, for the birds wintering off Asia, a very long and rapid return migration. Similar patterns of rapid movements among discrete, distantly spaced feeding areas are visible in the migrations of some other seabirds (e.g. Short-tailed Albatross *Phoebastria albatrus* [37], Manx Shearwaters *Puffinus puffinus* [38], Arctic Tern *Sterna paradisea* [39], Northern Gannet *Sula bassanus* [34]) where detailed movement histories are available and such have been inferred from distributional data for a wide range of species [40]. Such patterns may be characteristic of many long-distance migrant seabirds.

Cargo ships, including tankers, arriving at or departing from ports on the mainland coast of northern British Columbia (Kitimat, Prince Rupert) reach international waters either via Hecate Strait, or via Dixon Entrance, while substantial traffic also passes up and down Hecate Strait and the eastern half of Queen Charlotte Sound between ports on Vancouver Island and Prince Rupert [41]. The great circle route from the Juan de Fuca Strait (originating in Vancouver and Puget Sound) passes west of Haida Gwaii. A Voluntary Tanker Exclusion Zone keeps most tanker traffic between Alaska and the US west coast well offshore of Haida Gwaii, but other vessels may skirt close to the coast. Vessel traffic to the west of Haida Gwaii is much less concentrated than in Queen Charlotte Sound and Hecate Strait. Consequently, the likelihood of accidents or spills occurring in northern BC waters affecting family parties of Ancient Murrelets following colony departure will differ among colonies depending on their dispersal after departure.

The numbers of birds breeding on the east and west coasts of Haida Gwaii are roughly similar [17], hence the number of birds at risk during the brood-rearing period would be comparable. Birds from the west coast colonies dispersed offshore more rapidly than those from the east coast colonies. By the fourth week after colony departure the average positions of birds from west coast colonies were all >200 km north and west from the NW tip of Haida Gwaii, whereas the average positions of birds from the east coast colonies which had not crossed 140° W were all within 150 km of the southern tip of Haida Gwaii and more importantly, most were in continental shelf waters of Queen Charlotte Sound. Even in the sixth week after departure, eight of the twelve tagged birds still remaining east of 140° W were in SE Hecate Strait or Queen Charlotte Sound. Consequently, the period of vulnerability to oil spills in Canadian waters for family parties from east coast colonies is roughly double that for the west coast colonies. Routes passing through Queen Charlotte Sound therefore pose a greater threat to Ancient Murrelet families than those passing through Dixon Entrance. Finally, a third of the birds moved back to winter in waters off western N America, including northern British Columbia. Hence, some Haida Gwaii breeders are vulnerable to oil spills in marine waters off northern British Columbia from November onwards.

## Summary

The previous characterisation of Ancient Murrelet movements as being without clearly defined wintering areas [1] was incorrect and illustrates how misleading “reasonable assumptions” may be. It is becoming clear from this and similar tracking studies that the movements of marine birds are both more complex and more precise than we had previously imagined. The existence of significant concentrations in non-breeding areas means an elevated risk to populations from point-source events, such as oil spills, and reinforces the frequent assertion that additional information on non-breeding movements and concentrations is highly desirable for better management of marine birds [16].
